# Caffeic Acid Phenethyl Ester Causes p21^Cip1^ Induction, Akt Signaling Reduction, and Growth Inhibition in PC-3 Human Prostate Cancer Cells

**DOI:** 10.1371/journal.pone.0031286

**Published:** 2012-02-07

**Authors:** Hui-Ping Lin, Shih Sheng Jiang, Chih-Pin Chuu

**Affiliations:** 1 Institute of Cellular and System Medicine, National Health Research Institutes, Miaoli, Taiwan; 2 Translational Center for Glandular Malignancies, National Health Research Institutes, Miaoli, Taiwan; 3 National Institute of Cancer Research, National Health Research Institutes, Miaoli, Taiwan; 4 Graduate Program for Aging, China Medical University, Taichung, Taiwan; Florida International University, United States of America

## Abstract

Caffeic acid phenethyl ester (CAPE) treatment suppressed proliferation, colony formation, and cell cycle progression in PC-3 human prostate cancer cells. CAPE decreased protein expression of cyclin D1, cyclin E, SKP2, c-Myc, Akt1, Akt2, Akt3, total Akt, mTOR, Bcl-2, Rb, as well as phosphorylation of Rb, ERK1/2, Akt, mTOR, GSK3α, GSK3β, PDK1; but increased protein expression of KLF6 and p21^Cip1^. Microarray analysis indicated that pathways involved in cellular movement, cell death, proliferation, and cell cycle were affected by CAPE. Co-treatment of CAPE with chemotherapeutic drugs vinblastine, paclitaxol, and estramustine indicated synergistic suppression effect. CAPE administration may serve as a potential adjuvant therapy for prostate cancer.

## Introduction

Prostate cancer is one of the most common non-cutaneous carcinoma of men in western countries. More than 80% of patients died from prostate cancer developed bone metastases [1]–[3]. In 1941, Charles Huggins discovered that deprivation of androgen caused regression of hormone-responsive metastatic prostate cancer [4]. Since then, androgen ablation therapy has become the primary treatment for metastatic prostate cancer. However, most prostate cancer patients receiving androgen ablation therapy ultimately develop recurrent, castration-resistant tumors within 12–33 months after treatment. The median overall survival time is 1–2 years after tumor relapse [5], [6]. Chemotherapy is usually applied for treatment of metastatic hormone-refractory prostate cancer [7].

Commonly used chemotherapy drugs for metastatic prostate cancer include eoposide, paclitaxol, vinblastine, mitoxantrone, and estramustine. Etoposide and mitoxantrone are type II topoisomerase inhibitor [7], [8]. Estramustine is a derivative of estrogen with a nitrogen mustard-carbamate ester moiety [7]. Vinblastine binds tubulin and inhibits assembly of microtubules [7]. Paclitaxel disrupts mitotic spindle assembly, chromosome segregation, and cell division. Paclitaxel also stabilizes the microtubule polymer and thus protects it from disassembly [7]. Treatment with these chemotherapy drugs decreased prostate specific antigen (PSA) and radiographic response as well as improved pain and urinary symptoms in a sub-group of patients. However, they showed little effect on prolonging survival. Undesired side effects of these chemotherapeutic agents include toxic deaths, strokes, thrombosis, neutropenia, edema, dyspnea, malaise, and fatigue [7]. Co-treatment chemotherapy drugs with natural compounds with anticancer activity may reduce the dosage of chemotherapy drugs needed.

Caffeic acid phenethyl ester (CAPE), a bioactive component extracted from honeybee hive propolis, is a strong antioxidant [9], [10]. CAPE treatment in breast, prostate, and leukemic cancer cells causes inhibition of NF-κB activity [11], [12], induction of Bax [11], [13], activation of c-Jun N-terminal kinase (JNK) [11] and p38 mitogen-activated protein kinase (p38 MAPK) [11]. CAPE induces apoptosis via activation of caspase activity [11], [13] and down-regulation of Bcl-2, cIAP-1, cIAP-2, and XIAP [12], [13] in breast, prostate, and leukemic cancer cells. In addition, CAPE induces cell cycle arrest through suppression of cyclin D1 [14], [15], cyclin E [14], and c-Myc expression [15], as well as increases expression of the cyclin dependent kinase inhibitors p21^cip1^ [14], p27^Kip1^ [14], and p16^INK4A^ [14] in colon and glioma cancer cells. These observations suggest that CAPE is a potential therapeutic agent for cancers.

PC-3 is one of the most commonly used prostate cancer cell lines established from bone-derived metastases. PC-3 cells do not express androgen receptor (AR) [16]. Mitoxantrone, estramustine, vinblastine, etoposide, and paclitaxel have been shown to induce proliferation inhibition, apoptosis, and cell cycle arrest in PC-3 cells *in vitro* [17]–[21], as well as to retard PC-3 xenografts growth in athymic nude mice [8], [21], [22]. Treatment with 88–176 µM of CAPE induced apoptosis in PC-3 cells via inhibition of NF-κB, cIAP-1, cIAP-2, and XIAP [12]. However, the achievable concentration of CAPE in human serum is around 5.0 µg/ml (17 µM) [23]. We thus examined if low concentration (0–20 µM) of CAPE can suppress the proliferation of PC-3 cells. We also determined if co-treatment of chemotherapy drugs with CAPE show synergistic inhibition effect on proliferation of PC-3 cells.

## Results

### CAPE treatment suppresses the proliferation and colony formation of PC-3 cells

**Figure 1:**
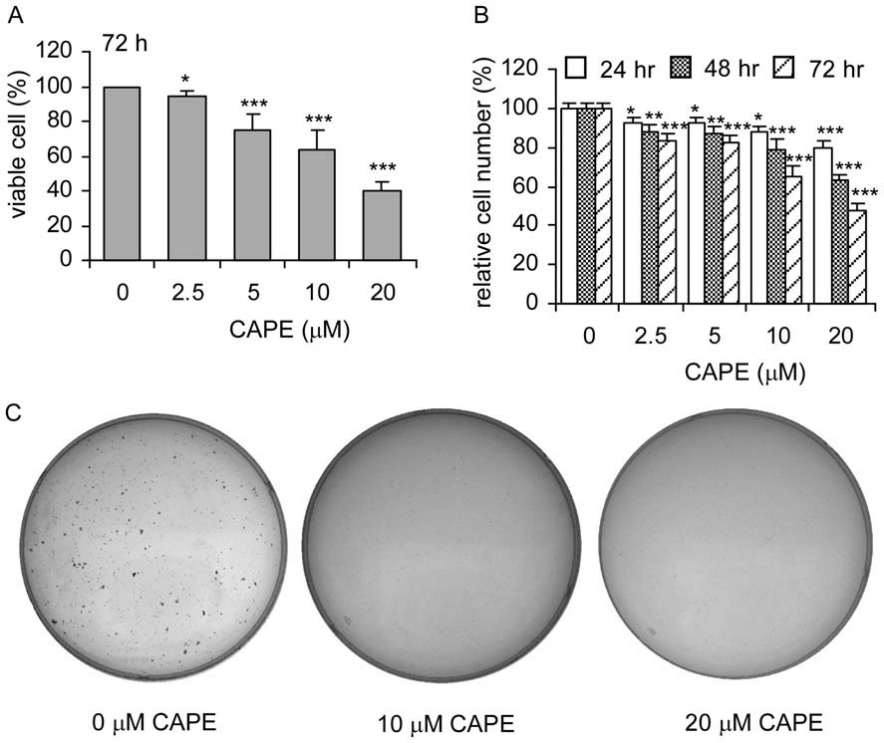
CAPE suppresses proliferation and colony formation of PC-3 cells. Proliferation of PC-3 cell treated with increasing concentration of CAPE was determined by Trypan blue staining after 72 h treatment (A) or measuring total DNA content per well using Hoechst 33258 fluorescence by 96-well proliferation assay after 24, 48, and 72 h treatment (B). Relative cell numbers were normalized to the average cell number of the control (no CAPE treatment) of each cell line in each individual experiment. Columns represent mean for 18 replicates; bars represent standard deviation. Asterisk (*) represents cell number is statistically significantly different (*p*<0.05) compared to the control. Columns represent mean for 5 biological replicates; bars represent standard deviation. (C) Anticancer effect of CAPE was determined by colony formation assay of PC-3 cells treated with 0, 10, 20 µM for 14 days. Image is a representative result of three biological replicates.

Trypan blue staining indicated that CAPE dose-dependently inhibited proliferation of PC-3 cells with an EC_50_ around 20.4 µM (Fig. 1A). Hoescht dye-based 96-well proliferation assay showed that the growth inhibitory effect of CAPE happened within 24 hours following CAPE treatment at concentration as low as 2.5 µM (Fig. 1B). EC_50_ for growth inhibition of PC-3 cells was 51.4 µM, 30.7 µM, and 23.1 µM for 24, 48, and 72 h CAPE treatment, respectively, indicating that the suppressive effect of CAPE can be accumulated. Colony formation assay revealed that treatment of 10 µM and 20 µM CAPE efficiently inhibited the formation of PC-3 colonies in soft agar (Fig. 1C).

**Figure 2:**
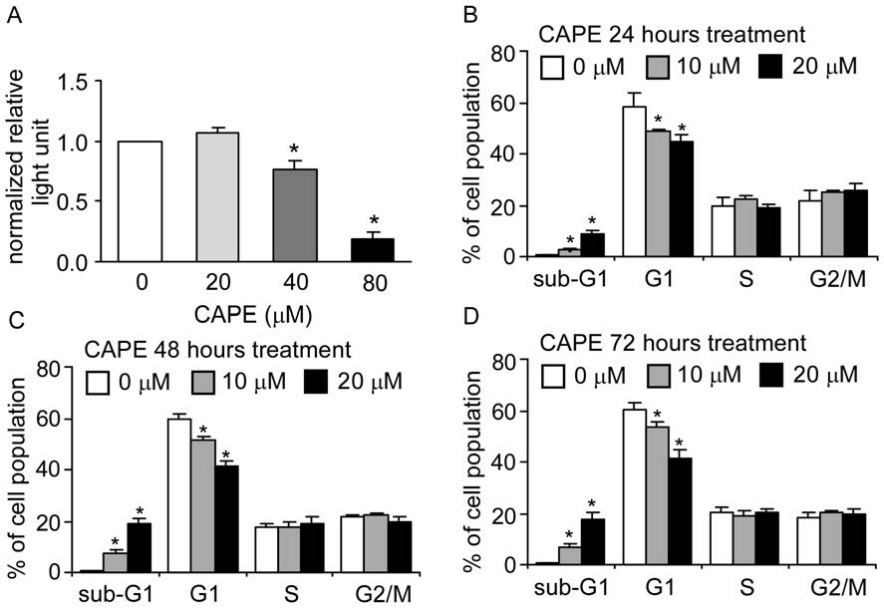
CAPE inhibits cell cycle progression in PC-3 cells. (A) PC-3 cells transfected with a 4X NF-κB luciferase reporter plasmid for 24 hr were treated with increasing concentrations of CAPE for additional 24 h. Relative luciferase activity was determined to compare the effect of CAPE on NF-κB transcriptional activity. (B) PC-3 cells were treated with CAPE for 24, 48, or 72 h, harvested, and stained with propidium iodide dye for flow cytometric analysis for cell cycle distribution. (*) represents statistically significant difference (*p*<0.05) between the two group of cells being compared.

Since CAPE was previously reported as an NF-κB inhibitor [10], we determined whether low dasage of CAPE can inhibit NF-κB activity using a plasmid-based luciferase reporter assay. Although CAPE treatment at 40 µM inhibited NF-κB activity, treatment with CAPE at concentration lower than 40 µM had no effect on NF-κB activity (Fig. 2A). This observation suggested that other mechanisms are responsible for CAPE's inhibitory effect at low dosage.

### CAPE treatment disturbs cell cycle progression

**Figure 3:**
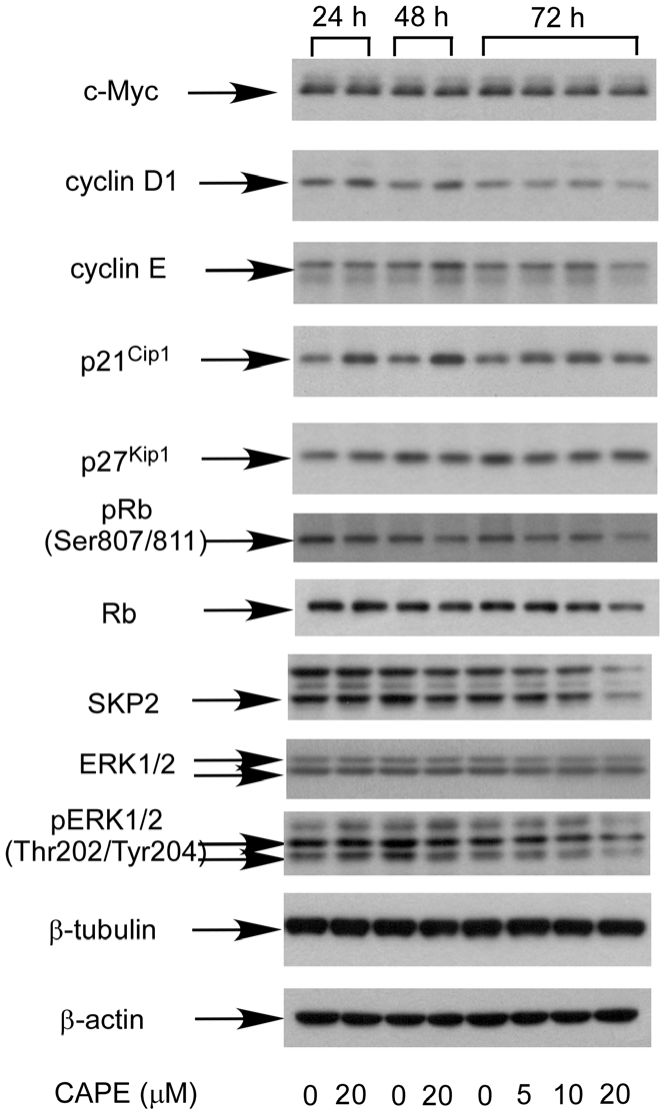
CAPE affects cell cycle regulating proteins in PC-3 cells. Protein expression of c-Myc, cyclin D1, cyclin E, SKP2, phosho-Rb (S807/811), p27^Kip1^, p21^Cip1^, ERK1/2, pERK1/2 Thr202/Tyr204, β-tubulin, and β-actin in PC-3 cells treated with 20 µM CAPE for 24, 48, and 5, 10, 20 µM CAPE for 72 h were assayed by Western blotting.

Propidium iodide (PI) staining flow cytometry analysis revealed that treatment with 10–20 µM CAPE decreased the cell population in G1 phase and increased cell population in sub-G1 phase within 24 h in PC-3 cells. This effect was more dramatic at 72 h following CAPE treatment (Fig. 2B–2D). However, annexin V staining flow cytometry analysis indicated that 10–20 µM CAPE did not induce apoptosis in PC-3 cells (data not shown). Treatment with 20 µM CAPE for 72 h resulted in increase of cell cycle inhibitory proteins p21^Cip1^ and decrease of S-phase kinase-associated protein 2 (SKP2), phosphorylation of serine 807/811 on retinoblastoma (Rb), cycin D1, cyclin E, c-Myc, and phosphorylation of threonine 202/tyrosine 204 of extracellular signal-regulated kinase 1/2 (ERK1/2) (Fig. 3). No change in p27^Kip1^, total ERK1/2, or β-tubulin was observed. Compared to 24 h and 48 h treatment, 72 h treatment in general caused more change of protein expression level except for cyclin D1. This may explain the greater growth inhibition caused by CAPE at 72 h. Cyclin D1 increased after 24 h and 48 h treatment but decreased after 72 h treatment.

### CAPE treatment inhibits the abundance and activity of proteins in AKT-signaling pathway

**Figure 4:**
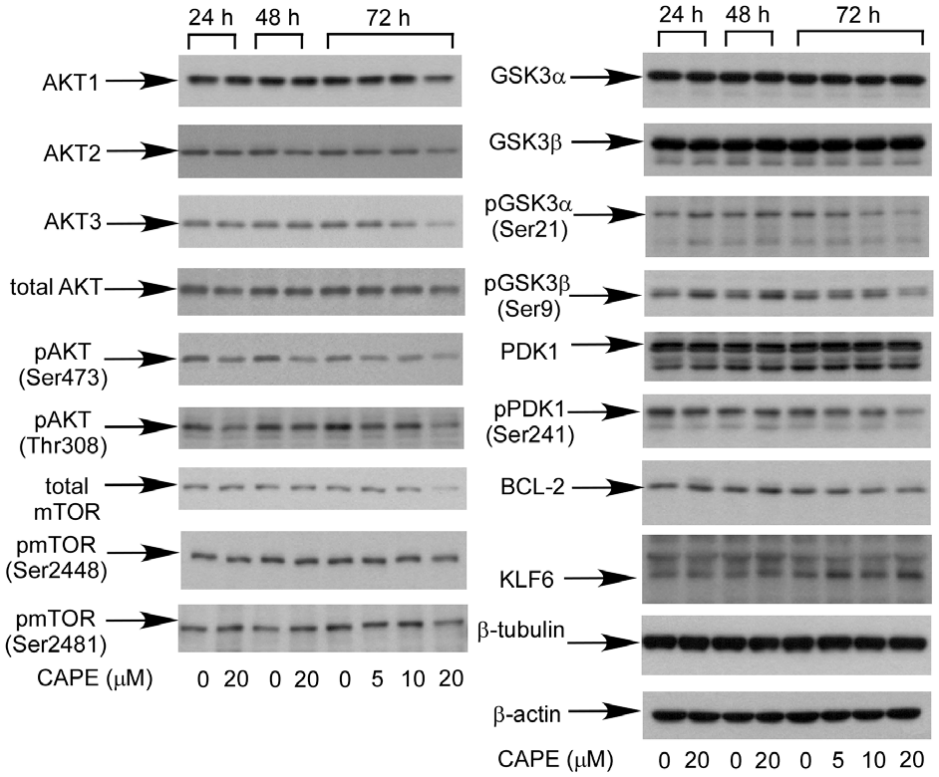
CAPE inhibits Akt signaling-related proteins in PC-3 cells. Protein expression of Akt, Akt1, Akt2, Akt3, total Akt, phospho-Akt S473, phospho-Akt T308, mTOR, phospho-mTOR Ser2448 and Ser2481, GSK3α, GSK3β, phopho-GSK3α S21, phospho-GSK3β S9, PDK1, phospho-PDK1 Ser241, Bcl-2, KLF6, β-tubulin, and β-actin in PC-3 cells treated with 20 µM CAPE for 24, 48, and 5, 10, 20 µM CAPE for 72 h were assayed by Western blotting.

Akt plays important role in survival and proliferation of prostate cancer cells [24]. We thus determined if CAPE treatment suppresses Akt signaling pathway. 72 h after 20 µM CAPE treatment decreased the abundance of total Akt, Akt1, Akt2, and Akt3 (Figure 4). CAPE treatment for 24–72 h significantly decreased the phosphorylation of Akt on serine 473 and threonine 308,. CAPE did not change the total abundance of phosphoinositide dependent kinase 1 (PDK1) (Fig. 4), however, phosphorylation of serine 241 on PDK1 was reduced by CAPE treatment. CAPE treatment also caused decrease of total mammalian target of rapamycin (mTOR) and slight reduction of phosphorylation on serine 2448 and 2481 of mTOR. CAPE treatment did not change the total abundance of GSK3α and GSK3β (Fig. 4). However, phosphosphorylation of GSK3α S21 and GSK3β S9 was increased after 24 h and 48 h of 20 µM CAPE treatment but decreased at 72 h of 20 µM CAPE treatment (Fig. 4). Bcl-2 is an anti-apoptosis factor downstream of Akt signaling. Overexpression of Bcl-2 has previously been reported to confer drug resistance of prostate cancers [5]. CAPE slightly decreased expression of Bcl-2.

### CAPE treatment affects genes regulating proliferation, survival, and death of PC-3 cells

**Figure 5:**
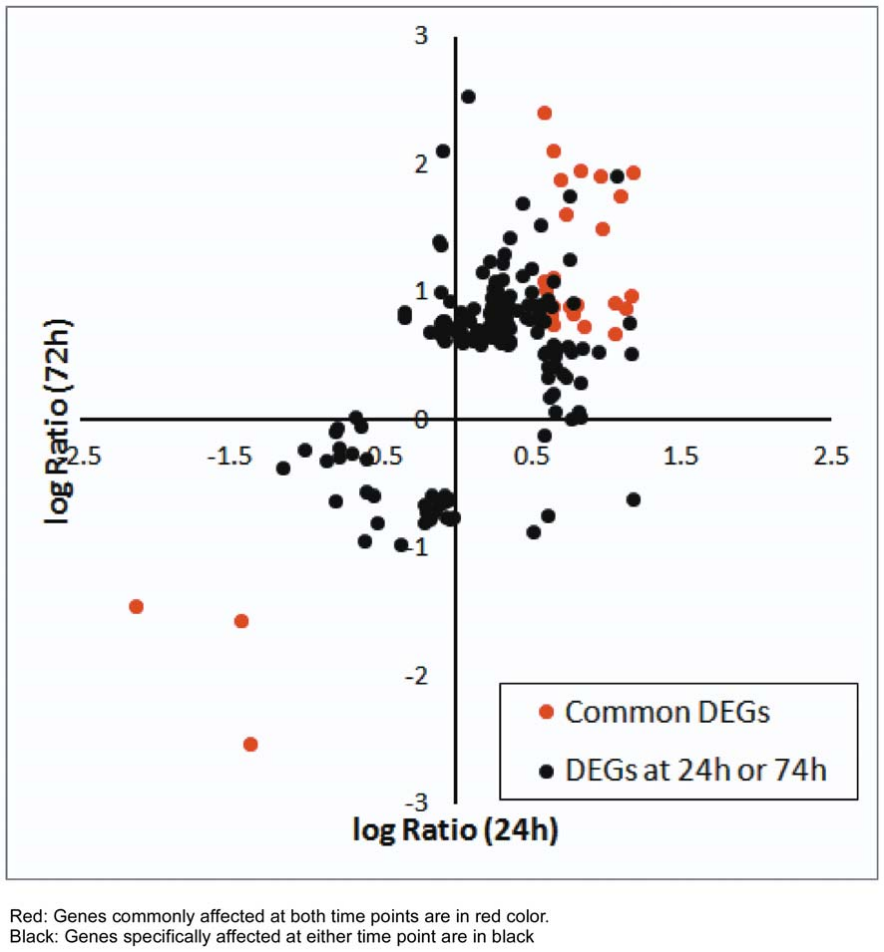
A scatter plot of log_2_ ratio (logR) for genes whose expression were significantly affected at either 24 h or 72 h post CAPE treatment. Genes commonly affected at both time points are in red color, while those specifically affected at either time point are in black. IPA analysis of the unique genes (n = 191) genes changed either in 24 h or 72 h CAPE treatment indicated that group of genes regulating several cell functions, including cell proliferation (p-value 9.82×10^−11^, 52 genes), cell growth (p-value 1.40×10^−10^, 41 genes), cell death (p-value 1.40×10^−12^, 68 genes), and cell survival (p-value 1.40×10^−6^, 27 genes).

We further studied the comprehensive change of gene expression in PC-3 cells treated with 20 µM CAPE for 24 h or 72 h by microarray. Genes with expression fold change >1.5 and *P*<0.05 were considered as genes significantly affected by CAPE treatment. CAPE affected expression of 69 unique genes after 24 h treatment (Table S1). 53 genes were up-regulated and 16 genes were down-regulated. Treatment with CAPE for 72 h altered expression of 147 unique genes (Table S2). 122 genes were up-regulated while 25 genes were down-regulated. 25 genes were commonly changed in both 24 h and 72 h treatment (Figure 5, Table S3). Among the 25 genes, 3 genes were down-regulated (CYP1B1, SCG5, PADI4) and 22 genes were up-regulated (LY96, LOC728285, TM4SF19, RGS2, PI3, AKR1C2, GDF15, HIST1H2BD, CCL20, CXCL5, RND3, KRT34, HIST2H2AA3, AKR1C4, KLF4, DUSP5, NOV, GK, CDKN1A, CXCL2, DUSP1, and HIST1H4H) (Fig. 5). Analysis of all the 191 gene probes affected by CAPE treatment either at 24 h or 72 h using Ingenuity Pathway Analysis (IPA) revealed that CAPE treatment affected genes involved in regulation of cell death, proliferation, and survival. Among the genes being affected by CAPE treatment, 52 genes involved in cell proliferation regulation (*p value* = 9.82×10^−11^), 41 genes involved in cell growth regulation (*p value* = 1.40×10^−10^), 68 genes involved in cell death regulation (*p value* = 1.40×10^−12^), and 27 genes involved in cell survival regulation (*p* = 3.43×10^−6^). Complete list of genes probes involved in these signaling pathways were shown in Table S4.

**Figure 6:**
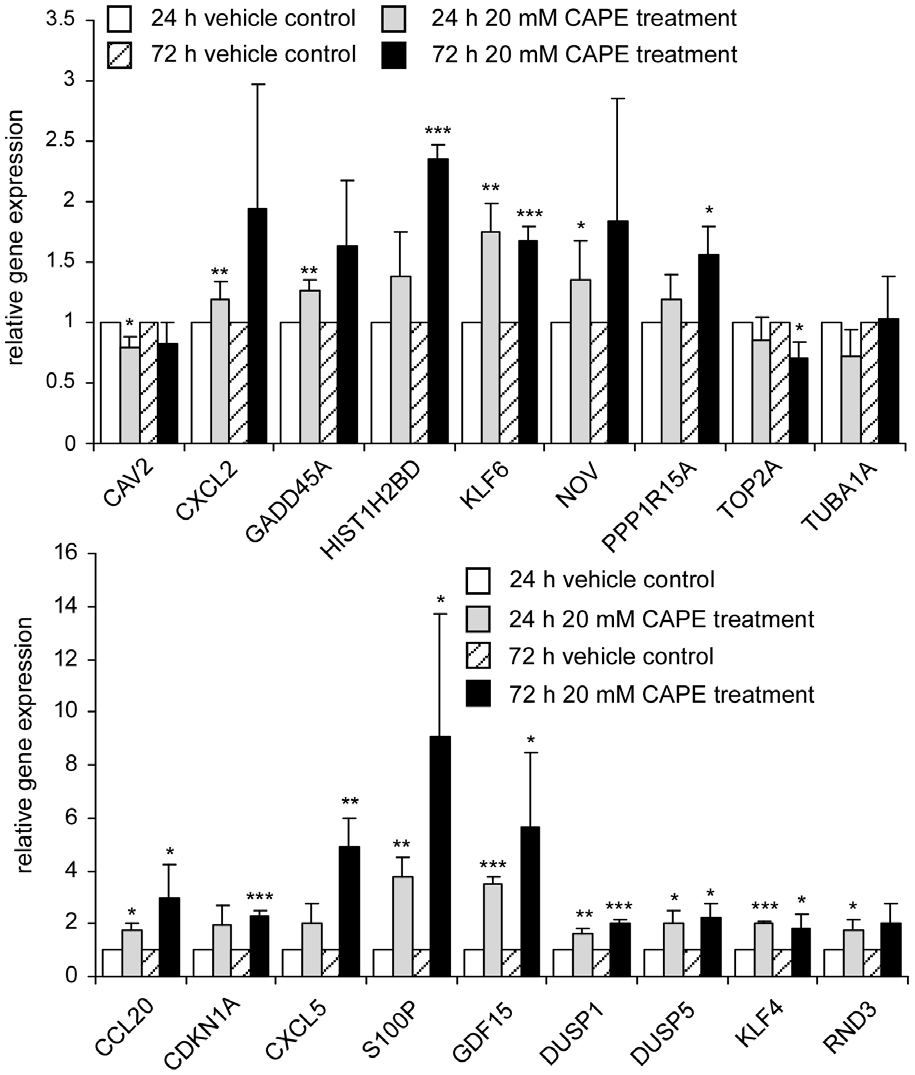
Validation of gene microarray result with qRT-PCR. Gene expression level of GDF15, HIST1H2BD, CCL20, CXCL5, RND3, KLF4, DUSP5, NOV, CDKN1A, CXCL2, DUSP1, KLF6, TOP2A, PPP1R15A, CAV2, S100P, GADD45A, and TUBA1A in PC treated with 0 or 20 µM CAPE for 24 h or 72 h was determined by qRT-PCR.

We validated some of the genes affected by CAPE treatment with quantitative real-time PCR (qRT-PCR). 17 out of 18 genes (GDF15, HIST1H2BD, CCL20, CXCL5, RND3, KLF4, DUSP5, NOV, CDKN1A, CXCL2, DUSP1, KLF6, TOP2A, PPP1R15A, CAV2, S100P, and GADD45A) tested by qRT-PCR showed similar alteration pattern following 24 h or 72 h CAPE treatment as compared to gene microarray. The only exception is TUBA1A. We did not observe any change of TUBA1A gene under CAPE treatment by qRT-PCR (Fig. 6). Western blotting assay indicated that protein level of KLF6 was increased by CAPE treatment (Fig. 4).

### Co-treatment of CAPE with chemotherapeutic drugs suppressed proliferation of PC-3 cells

**Figure 7:**
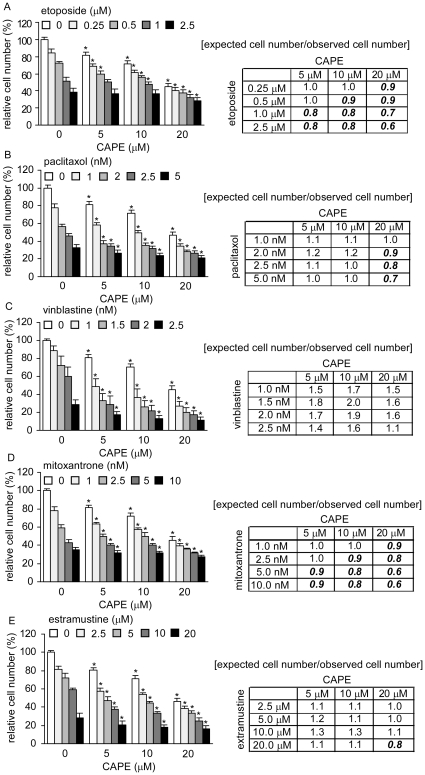
Combined treatment of CAPE with chemotherapy drugs shows synergistic and antagonistic inhibition on proliferation of PC-3 cells. Proliferation of PC-3 cells treated with increasing dosage (0, 5, 10, 20 µM) of CAPE in combination with increasing concentration of etoposide (A), paclitaxol (B), vinblastine (C), mitoxantrone (D), and estramustine (E) for 72 h was determined by 96-well proliferation assay. The right part of the figure show the ratio of expected cell number/observed cell number. For example, treatment of 5 µM of CAPE or 1 nM vinblastine decreases cell number of PC-3 to 80.9% and 88.7%, respectively, compared to the control (no treatment). The expected cell number of treatment combining 5 µM of CAPE and 1 nM vinblastine is 0.809*0.887 = 71.8%. The observed cell number is 48.8% compared to the control. So the ratio is 0.718/0.488 = 1.5. Ratio larger than one represents synergy of growth inhibition, while ratio smaller than one represents antagonistic effect.

Finally, we investigated if co-treatment of CAPE at serum-available dosage (0–20 µM) with commonly used chemotherapy drugs (etoposide, paclitaxol, vinblastine, mitoxantrone, and estramustin) can suppress growth of PC-3 cells more effectively than treatment with chemotherapy drugs alone. EC_50_ of CAPE, etoposide, paclitaxol, vinblastine, mitoxantrone, and estramustin for inhibiting proliferation of PC-3 cells was 18.3 µM, 1.7 µM, 3.0 nM, 2.1 nM, 5.9 nM, and 13.0 µM. Treatment of 20 µM CAPE suppressed growth of PC-3 cells more effectively than treatment with 1.0 µM etoposide, 2.5 nM paclitaxol, 5 nM mitoxantrone, 2 nM vinblastine, or 10 µM estramustine (Figure 7). When co-treatment with 20 µM CAPE, 0.5 µM etoposide, 1 nM paclitaxol, 1 nM vinblastine, 2.5 nM mitoxantrone, and 8 µM extramustine caused growth inhibition similar to the highest dosage we tested (Figure 7). Synergistic effect means the suppressive effect of two drugs being treated together is greater than the sum of their separate suppressive effect at the same doses, while antagonistic effects means the suppressive effect of two drugs is less than the sum of the effect of the two chemicals taken separately. According to the definition, co-treatment of CAPE showed synergistic effect with vinblastine, estramustine, or paclitaxol, and antagonistic effect with etoposide or mitoxantrone (Figure 7).

**Figure 8:**
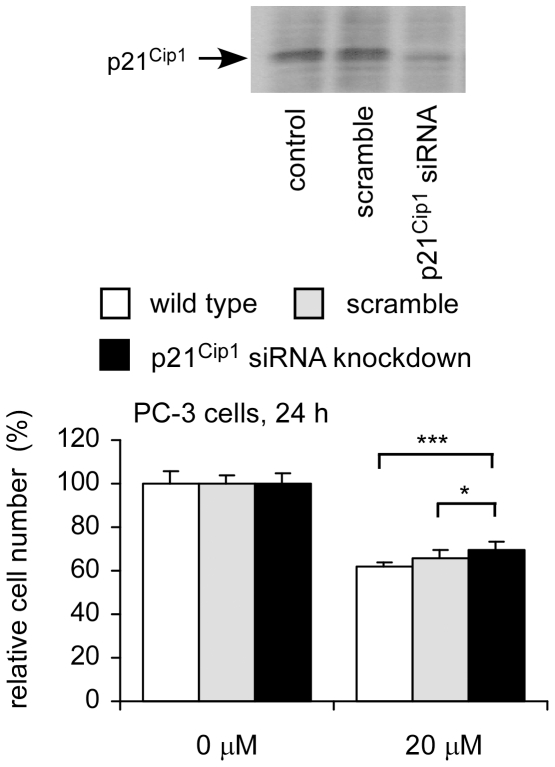
Growth response to CAPE treatment of PC-3 and PC-3 p21^Cip1^ siRNA cells. Protein levels of wild type PC-3, PC-3 cells transfect with scramble control (20 nM), and PC-3 cells transfected with p21^Cip1^ siRNA (20 nM) were determined by Western blotting assay. Proliferation of these PC-3 cells treated with 20 µM CAPE for 24 h was determined by 96-well plate proliferation assay as described in Material and Methods.

According to our observation, p21^Cip1^ plays important role in regulation of growth inhibition induced by CAPE treatment. To confirm this, we knocked down p21^Cip1^ in PC-3 and determined if these PC-3 cells become more resistant to CAPE treatment. As expected, following 24 h of CAPE treatment, PC-3 cells with less p21^Cip1^ protein expression were more resistant to growth inhibition caused by CAPE treatment (Fig. 8).

## Discussion

Our observation suggested that caffeic acid phenethyl ester (CAPE) can inhibit proliferation and colony formation of PC-3 human prostate cancer cells at concentration 10–20 µM. These observations suggested that the achievable concentration of CAPE in human serum, (17 µM) [23], is possibly to cause growth inhibition in prostate tumors in patients.

Cyclin-dependent kinase inhibitor p21^Cip1^ binds and inhibits the kinase activities of Cdk2/cyclin A, Cdk2/cyclin E, Cdk4/cyclin D, and Cdk6/cyclin D complexes [25]. p21^Cip1^ can interact with proliferating cell nuclear antigen (PCNA), a DNA polymerase accessory factor, and plays a regulatory role in S phase DNA replication and DNA damage repair [26]. SKP2 is a member of the F-box protein family. SKP2 constitutes one of the four subunits of ubiquitin protein ligase complex called SCFs (SKP, cullin, F-box containing complex), which functions in phosphorylation-dependent ubiquitination. SKP2 is an essential element of the cyclin A-Cdk2 S-phase kinase [27]. Reduction in phosphorylation of Rb restricts cell proliferation by inhibiting E2F activity [28]. ERK1 and ERK2 are involved in the control of many fundamental cellular processes including cell proliferation, survival, differentiation, apoptosis, motility and metabolism. ERK1/2 play important roles in canonical MAPK (Mitogen-Activated Protein Kinase) signaling pathway and are critical regulators of the growth and survival [29]. CAPE induced p21^Cip1^ and reduced cyclin D, cyclin E, SKP2, and phosphorylation of Rb and ERK1/2 (Fig. 3). CAPE may thus suppress the growth of PC-3 cells via these proteins [30].

Akt is a serine/threonine protein kinase regulating inhibition of apoptosis and stimulation of cell proliferation. Up-regulation of PI3K/Akt activity is associated with poor clinical outcome of prostate cancer [31]. There are three mammalian isoforms of this enzyme, Akt1, Akt2, and Akt3 [32], [33]. Protein abundance and activity of Akt3 have previously been suggested to contribute to the more aggressive clinical phenotype of androgen non-responsive prostate and breast cancers [34]. Akt3 enzymatic activity was approximately 20-60-fold higher in AR-negative PC-3 and DU-145 cells compared to the AR-positive LNCaP prostate cancer cells [34], [35]. We observed that CAPE suppressed Akt signaling-related proteins, including Akt1, Akt2, Akt3, total Akt, mTOR, Bcl-2, pAkt Ser 473, pAKt Thr 308, pmTOR Ser 2448/2481, pGSK3α Ser21, pGSK3β Ser9, and pPDK1 Ser241. CAPE was recently reported to suppress phosphorylation of Akt in other human cells, such as CD4+ T cells [36], coronary smooth muscle cell [37], and A549 lung cancer cells [38]. Phosphatase and tensin homolog (PTEN) protein acts as a phosphatase to dephosphorylate phosphatidylinositol (3,4,5)-trisphosphate. PTEN negatively controls the phosphoinositide 3-kinase/Akt signaling pathway [39]. PC-3 cells acquire a homozygous deletion of PTEN, thus Akt is constantly active. There are two phosphorylation sites on Akt, threonine 308 and serine 473. Phosphorylation of Thr308 on Akt is activated by PDK1 [40]. Phosphorylation of serine 473 is activated by mTOR kinase, its associated protein rector, and SIN1/MIP1 [41], [42]. CAPE phosphorylation of serine 241 on PDK1 and attenuated the phosphorylation of serine 2448 and 2481 on mTOR (Fig. 4). Reduction of PDK1 and mTOR activity may therefore contribute to the decrease of phsphorylation on Akt. The activities of glycogen synthase kinase 3 alpha (GSK3α and GSK3β are known to be inhibited by Akt-mediated phosphorylation at Ser21 and Ser9 respectively, limiting their ability to phosphorylate cell cycle regulating proteins, such as cyclin D1 and p21^Cip1^ [43], [44]. Phosphosphorylation of GSK3α S21 and GSK3β S9 was increased after 24 h and 48 h of 20 µM CAPE treatment but decreased at 72 h of 20 µM CAPE treatment (Fig. 4). Increased phosphorylation of GSK3α S21 and GSK3β S9 may contribute to the increase of p21^Cip1^ at 24 h and 48 h after CAPE treatment. GSK3β-dependent phosphorylation of cyclin D1 mediated nuclear export and rapid degradation within the cytoplasm of cyclin D1 [45]. Reduction of GSK3βactivity due to increase of phosphorylation (Fig. 4) resulted in less phosphorylation of cyclin D1 and therefore accumulation of cyclin D1 at 24 h and 48 h (Fig. 3). Increase of GSK3βactivity due to decrease of phosphorylation (Fig. 4) would therefore decrease the abundance of cyclin D1 at 72 h (Fig. 3). Decreased phosphorylation of GSK3α and GSK3β at 72 h was consistent with the decreased phosphorylation of Akt. Suppression of Akt signaling by CAPE may contribute to the inhibition of survival and growth in PC-3 cells.

We noticed that genes affected by CAPE at 24 h and 72 h post treatment was moderately correlated (*r = *0.56, Fig. 5). There were only 25 significantly affected genes in common between these two time points. Since the growth inhibition and cell cycle perturbation caused by CAPE treatment started within 24 h and the suppressive effect accumulated over time, we hypothesized that the most important target genes for anticancer activity of CAPE were these 25 common genes. Krüppel-like factor 4 (KLF4) transactivates the p21^Cip1^ promoter and inhibits proliferation through activation of p21^Cip1^ as well as direct suppression of cyclin D1 and cyclin B1 gene expression [46]–[48]. Nov gene encodes protein CCN3 (Nov) which inhibits cell proliferation via Notch/p21^Cip1^ pathway [49]. Elevation of KLF4 and Nov genes may suppress PC-3 growth via p21^Cip1^. Growth/differentiation factor-15(GDF-15) is a divergent TGFβ family member that has been implicated in inhibition of tumor growth and increased tumor invasiveness [50]. A few genes are cytokines involved in inflammation response, such as CCL20 [51], CXCL2 [52], CXCL5 [53]. They were found up-regulated, suggesting that CAPE induces inflammation response in PC-3 cells. In addition, CAPE treatment increases RhoE/Rnd3. Up-regulation of the small G-protein RhoE/Rnd3/Rho8 inhibits the proliferation of prostate cancer cells by promoting apoptosis and inhibiting cell cycle progression [54].

Besides the 25 commonly changed genes, some differentially expressed genes specifically after 24 h or 72 h treatment also regulate cell survival, proliferation, or cell death. CAPE treatment increased KLF6, S100P, GADD45A, PPP1R15A, S100P, but decreased TOP2A and CAV2. Kruppel-like factor 6 (KLF6) is a zinc finger transcription factor and functions as tumor suppressor gene in human prostate cancer [55]. KLF6 up-regulates p21^Cip1^ in a p53-independent manner and significantly reduces cell proliferation [55]. S100P protein regulates calcium signal transduction, cytoskeletal interaction, protein phosphorylation, transcriptional control, cell cycle progression, and differentiation. Elevation of S100P in PC3 cells promoted cell growth, increased the percentage of S-phase cells, decreased basal apoptosis rate, promoted anchorage independent growth in soft agar, and confer resistance to chemotherapy [56]. GADD45A protein responds to environmental stresses by mediating activation of the p38/JNK pathway. The Gadd45 protein has been described to form a complex with p21^Cip1^. The p21^Cip1^-binding domain of GADD45A also encodes the Cdc2-binding activity. GADD45A interacts with Cdc2, dissociates the Cdc2-cyclin B1 complex, alters cyclin B1 nuclear localization, and thus inhibits the activity of Cdc2/cyclin B1 kinase [57]–[59]. PPP1R15A (Protein phosphatase 1 regulatory subunit 15A, also known as GADD34) has been shown to induce growth arrest and apoptosis. PPP1R15A up-regulation enhances p21^Cip1^ protein expression and induces p21^Cip1^ promoter activity [60].

**Figure 9:**
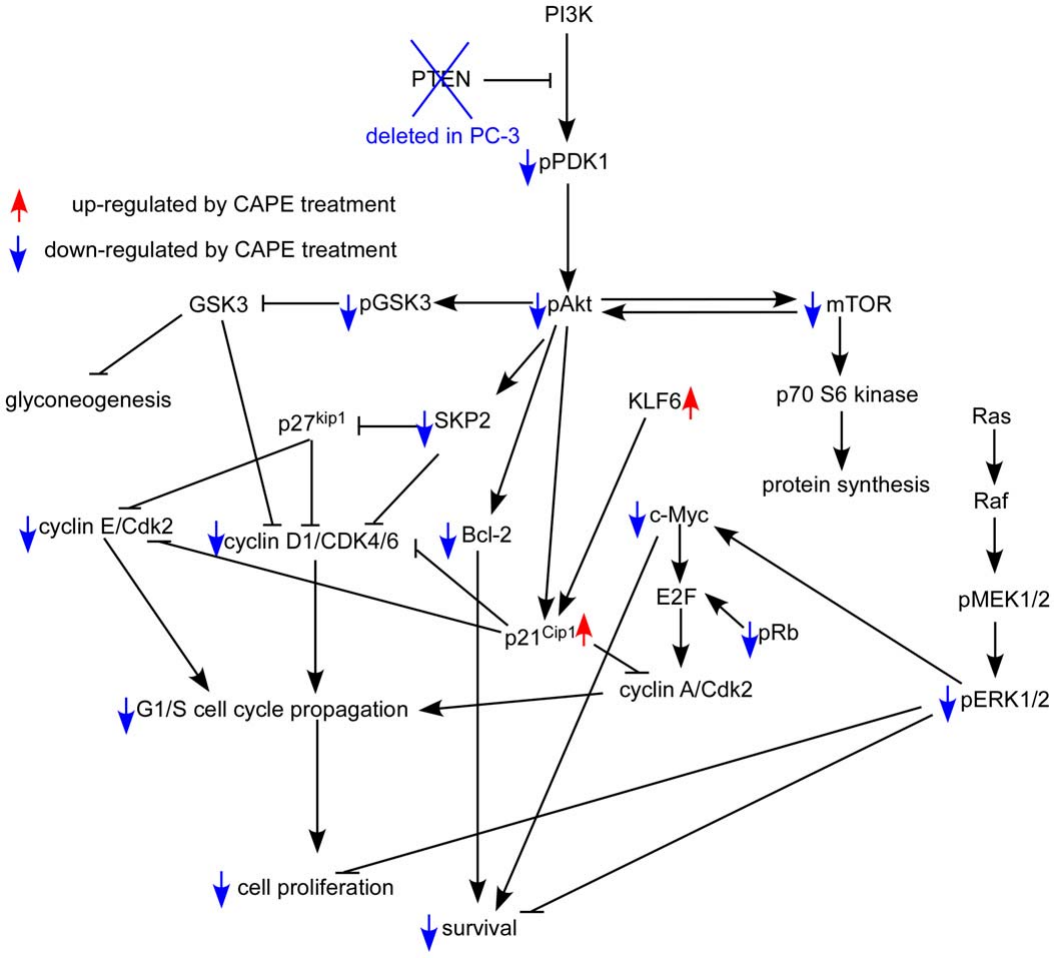
Putative model of anticancer effect of CAPE in PC-3 human prostate cancer cells. Protein abundance or activity being stimulated by CAPE treatment are labeled with red upward arrows, while those being suppressed by CAPE treatment are labeled with blue downward arrows.

Vinblastine, paclitaxol, and CAPE affect gene expression of α-tubulin and β-tubulin (Figure S1, S2), while etoposide, mitoxantrone, and CAPE affect gene expression of type II topoisomerase (Figure S3). However, etoposide induces p21^Cip1^ via p53 and down-regulation of c-Myc in cancer cells [61], [62]. Mitoxantrone induces p21^Cip1^ [63]. Vinblastine induces apoptosis via reduction of p21^Cip1^ [64]. Paclitaxol induces an Akt-dependent phosphorylation on p21^Cip1^ leading to an association of p21^Cip1^ with 14-3-3 and thus accumulation of the phosphorylated form of p21^Cip1^ in cytoplasm which prevents the inhibitory effect of p21^Cip1^ [65]. No study reports the relationship between p21^Cip1^ and estramustine. Since CAPE treatment increases both mRNA and protein level of p21^Cip1^(Fig. 3) and knockdown of p21^Cip1^ in PC-3 cells made cells more resistant to CAPE treatment (Fig. 8), CAPE may suppress growth and survival of PC-3 cells more similar to etoposide and mitoxantrone, but less similar to vinblastine, paclitaxol, and estramustine. Besides CDKN1A (p21^Cip1^ gene), CAPE treatment also increased gene expression of KLF4, KLF6, Nov, GADD45A, PPP1R15A. These genes all suppress proliferation via p21^Cip1^. Therefore, although co-treatment with CAPE suppressed more PC-3 cells than treatment with chemotherapy drug alone, CAPE only showed synergistic suppressive effect with vinblastine, paclitaxol, and estramustine (Fig. 7). CAPE treatment also suppressed abundance and phosphorylation of Akt, as well as upstream and downstream signaling proteins in Akt signaling. We therefore believe that p21^Cip1^ induction and suppression of Akt signaling both play important role in growth inhibition caused by CAPE treatment in PC-3 cells. We summarize the Akt/p21^Cip1^ signaling pathway network being affected by CAPE treatment in PC-3 in Figure 9.

In conclusion, our observations provided insight into the molecular mechanism of CAPE's anti-proliferative effect in PC-3 prostate cancer cells. Our data suggested that CAPE administration may be useful as a potential adjuvant therapy in combination with chemotherapies for metastatic prostate cancer.

## Materials and Methods

### Chemicals

Caffeic aicd phenethyl ester, etoposide, paclitaxol, vinblastine, estramustine, and mitoxantrone were purchased from Sigma (St. Louis, MO, U.S.A.).

### Cell Culture

PC-3 cells were generous gift from Dr. Shutsung Liao's lab (The University of Chicago) and were maintained in DMEM (Gibco/Invitrogen, Carlsbad, CA, U.S.A.) supplemented with 10% fetal bovine serum (FBS; Atlas Biologicals, Fort Collins, CO, U.S.A.), penicillin (100 U/ml), and streptomycin (100 ug/ml).

### Cell Proliferation Assay

Relative cell number was analyzed by measuring DNA content of cell lysates with the fluorescent dye Hoechst 33258 (Sigma) as described previously [66]–[69]. EC_50_ (concentration of drug to cause 50% growth inhibition) of drugs on PC-3 cells was determined by an Excel add-in program ED50V10.

### Soft Agar Colony Formation Assay

8000 cells were suspended in 0.3% low melting agarose (Lonza, Allendale, NJ, U.S.A.) with 10% fetal bovine serum in DMEM medium and then layered on top of 3 ml of 0.5% low melting agarose plus 10% fetal bovine serum in DMEM medium in 6 cm dishes. Cells were allowed to grow at 37°C with 5% CO_2_ for 14 days. The plates were stained with 0.005% crystal violet in 30% ethanol for 6 h.

### Luciferase-reporter Assay

PC-3 cells were seeded at 1.9×10^5^ cells/well in a 12-well plate in DMEM containing 10% FBS. 24 h after plating, PC-3 cells were transfected with pRL-TK-Renilla luciferase plasmid (normalization vector; 8 ng/well), 4X NF-κB (reporter gene vector; 800 ng/well) using the PolyJet™ in vitro DNA transfection reagent (SignaGen Laboratories, Rockville, MD). 24 h after transfection, cells were treated with increasing concentrations of CAPE. After an additional 24 hr, cells were lysed in 100 µL passive lysis buffer (Promega, Madison, WI, U.S.A.) and luciferase activity was measured using a Dual-Luciferase kit (Promega) in a 20/20^n^ luminometer Turner Biosystems.

### Flow Cytometric Analysis

Cells were seeded in 6 cm dishes in 4.5 mL of media and CAPE was added 24 h after plating. After indicated time (24, 48, 72 hours) of culture in the presence of various concentrations of CAPE, cells were removed with trypsin and fixed in 70% ethanol in PBS overnight at −20°C. Fixed cells were washed with PBS, treated with 0.1 mg/mL RNase A in PBS for 30 min, and then suspended in 50 µg/mL propidium iodide in PBS. Cell cycle profiles and distributions were determined by flow cytometric analysis of cells using a BD Facscan flow cytometer (BD Biosciences, San Jose, CA, U.S.A.) as previously described [69].

### Gene Microarray Analysis

Total RNAs were isolated from PC-3 cells treated with 20 µM CAPE or control vehicle for 24 or 72 hours using RNeasy mini kit (Qiagen, Valencia, CA, U.S.A.). The quantity of total RNA was determined by NanoDrop 2000 (Thermo Fisher Scientific, Waltham, MA, U.S.A.). The quality of total RNA samples were examined by Bioanalyzer 2100 (Agilent, Santa Clara, CA, U.S.A.) to avoid seriously degraded RNA. RNA samples with RNA integrity numbers (RIN) of <7 were excluded from this study. Complementary RNA targets were synthesized, amplified, labeled, and purified using the TargetAmp Nano-G Bioti-aRNA Labeling kit (Epicentre, Madison, WI, U.S.A.) according to the manufacturer's instruction [70]. Hybridization of labeled probe to Illumina BeadChips Human HT-12v3 was conducted according to protocol recommended by Illumina (San Diego, CA, U.S.A.). Each HT-12 chip has totally 48,804 unique 50-mer oligonucleotides probes with 15-fold feature redundancy in average [70]. Beadchips were scanned on the Illumina BeadArray 500GX reader and image processed by Illumina BeadScan software. Illumina BeadStudio software was used for preliminary data analysis [70]. All data is MIAME compliant and that the raw data has been deposited to the MIAMEExpress database (http://www.ebi.ac.uk/miamexpress/) (MIAMEExpress array databse accession number: E-MTAB-773).

### Western Blotting Analysis

Proteins were separated on 6–12% SDS-PAGE gels and expression levels were determined by Western blotting using following antibodies: Total Akt, Akt2, β-actin and PDK1 were from Novus (Littleton, CO, U.S.A.). Cyclin D1, Cyclin E, p-Akt (Ser 473), p-Akt (Thr 308), p-ERK1/2, GSK3α, GSK3β, p-GSK3α, p-GSK3β, mTOR, p-mTOR(Ser2481), p-PDK1(Ser241), Rb, and p-Rb(Ser807/811) were from Cell Signaling (Danvers, MA, U.S.A.). c-Myc was purchased from Epitomics (Burlingame, CA, U.S.A.). p21^Cip1^, p27^Kip1^ and SKP2 were purchased from Santa Cruz (Santa Cruz, CA, U.S.A.). KLF6 was from Abnova (Taipei, Taiwan). Akt1, Akt3, Bcl-2, ERK1/2, p-mTOR(Ser2448) and β-tubulin were from Millpore (Billerica, MA, U.S.A.). Anti-rabbit and anti-mouse IgG secondary antibodies were from Santa Cruz. β-actin was used as loading control.

### Quantitative real-time PCR

PC3 cells seeded in 10 cm dish were treated with 0 or 20 µM CAPE for 24 h or 72 h. Total RNA was isolated with RNeasy Mini Kit (Qiagen, Venlo, Hilden, Germany). cDNA was synthesized from total RNA using RevertAid H Minus First Strand cDNA Synthesis Kit (Fermentas, Waltham, Massachusetts, U.S.A.). Real-time PCR was performed on an ABI PRISM 7000 system (Applied Biosystems, Foster City, California, U.S.A.) using Maxima SYBR Green/ROX qPCR Master Mix (Fermentas). The sequences of primers are as following: CAV2 primers, 5′-agctgtctgcacatctggatt-3′(forward) and 5′-tcgtacacaatggagcaatga-3′(reverse); CCL20 primers, 5′- gaatcagaagcagcaagcaac-3′(forward) and 5′-cgtgtgaagcccacaataaat -3′(reverse); CDKN1A primers, 5′-caaaaactaggcggttgaatg-3′(forward) and 5′-aaaaggagaacacgggatgag-3′(reverse); CXCL2 primers, 5′-cttattggtggctgttcctga-3′(forward) and 5′-tcaaacacattaggcgcaatc -3′(reverse); CXCL5 primers, 5′- atctgcaagtgttcgccatag-3′(forward) and 5′-caaatttccttcccgttcttc-3′(reverse); DUSP1 primers, 5′-accatctgccttgcttacctt-3′(forward) and 5′-tgaagctgaagttgggagaga-3′(reverse); DUSP5 primers, 5′-ttgggtccaatgaggtagttg-3′(forward) and 5′-ccaaagtccaaggtcagtgaa-3′(reverse); GADD45A primers, 5′-gcagatggaaagaggtgaaaa-3′(forward) and 5′-agttttccttcctgcatggtt-3′(reverse); GDF15 primers, 5′-ctacaatcccatggtgctcat-3′(forward) and 5′-agtggcagtctttggctaaca-3′(reverse); HIST1H2BD primers, 5′-ggaagtctcatctgcctgaaa-3′(forward) and 5′-ttagttccttcccctcggtaa-3′(reverse); KLF4 primers, 5′-aagaacagatggggtctgtga-3′(forward) and 5′-ccttggcattttgtaagtcca-3′(reverse); KLF6 primers, 5′-taacggctgcaggaaagttta-3′(forward) and 5′-ccttcccatgagcatctgtaa-3′(reverse); NOV primers, 5′- ctctattggctccctttttgg -3′(forward) and 5′-ttgaagagctgcatgtttcct-3′(reverse); PPP1R15A primers, 5′-tgatgatgatggcatgtatgg-3′(forward) and 5′-ttatcagaaggctgggagaca-3′(reverse); RND3 primers, 5′-aagcggaacaaatcacagaga-3′(forward) and 5′-tcttcgctttgtcctttcgta-3′(reverse); S100P primers, 5′-gaaggcaggactcaaatgatg-3′(forward) and 5′-cctaggggaataattgccaac-3′(reverse); TOP2A primers, 5′-tgtcccagctctcatatttgg-3′(forward) and 5′-catttcgaccacctgtcactt-3′(reverse); TUBA1A primers, 5′-cttccaccctgagcaacttatc-3′(forward) and 5′-atctccttgccaatggtgtagt-3′(reverse).

### siRNA knockdown of p21^Cip1^

Human p21^Cip1^(CDKN1A) antisense and randomly scrambled sequence control were purchased from Thermo (Waltham, Massachusetts, U.S.A.). The transfection procedure was performed using lipofectamine RNAiMAX (Invitrogen, Carlsbad, CA, U.S.A.) according to the manufacturer's recommended protocal. 20 nM RNA were used for both scramble and p21^Cip1^ knockdown.

### Data Analysis

Data are presented as the mean +/− SD of at least three independent experiments. Student's t test (two-tailed, unpaired) was used to evaluate the statistical significance of results from proliferation assay experiments.

## Supporting Information

Figure S1 **A network enriched by IPA analysis with drug targets (TUBA) of docetaxel and vinblastine (colored in orange) indicated.** The union of differentially expressed genes (DEGs) at 24 h and 72 h post CAPE treatment was input to IPA. Upregulated genes are colored in red, and downregulated genes in green. Values of log ratio of expression change were also shown in the bottom of DEGs.
(JPG)

Figure S2 **A network enriched by IPA analysis with drug targets (beta tublin) of docetaxel and vinblastine (colored in orange) indicated.** The input of IPA analysis and its display is the same as in Figure S1.
(JPG)

Figure S3 **A canonical pathway (G2/M DNA damage checkpoint regulation) enriched by IPA analysis with drug targets (Topo II) of etoposide and mitoxantrone (colored in orange) indicated.** The input of IPA analysis and its display is the same as in Figure S1.
(JPG)

Table S1 **List of differentially expressed genes at 24 h post CAPE treatment.** Differentially expressed gene at 24 h post CAPE treatment was shown and value of these genes at 72 h post CAPE treatment was also shown for comparison.
(XLS)

Table S2 **List of differentially expressed genes at 72 h post CAPE treatment.** Differentially expressed gene at 72 h post CAPE treatment was shown and value of these genes at 24 h post CAPE treatment was also shown for comparison.
(XLS)

Table S3 **List of differentially expressed genes commonly appeared at 24 h and 72 h post CAPE treatment.** Expression of genes commonly changed at both 24 h and 72 h post CAPE treatment was shown.
(XLS)

Table S4 **IPA gene function ontology analysis of genes whose expression are significantly changed by CAPE treatment.** IPA gene function ontology analysis was shown of genes whose expression are significantly changed by CAPE treatment for 24 h and 72 h.
(XLS)
