# Supplementary figures and images for: Caffeic Acid Phenethyl Ester Causes p21Cip1 Induction, Akt Signaling Reduction, and Growth Inhibition in PC-3 Human Prostate Cancer Cells
Source: PLoS One. 2012 Feb 7;7(2):e31286. doi: 10.1371/journal.pone.0031286 (PMC3274546; doi:10.1371/journal.pone.0031286)

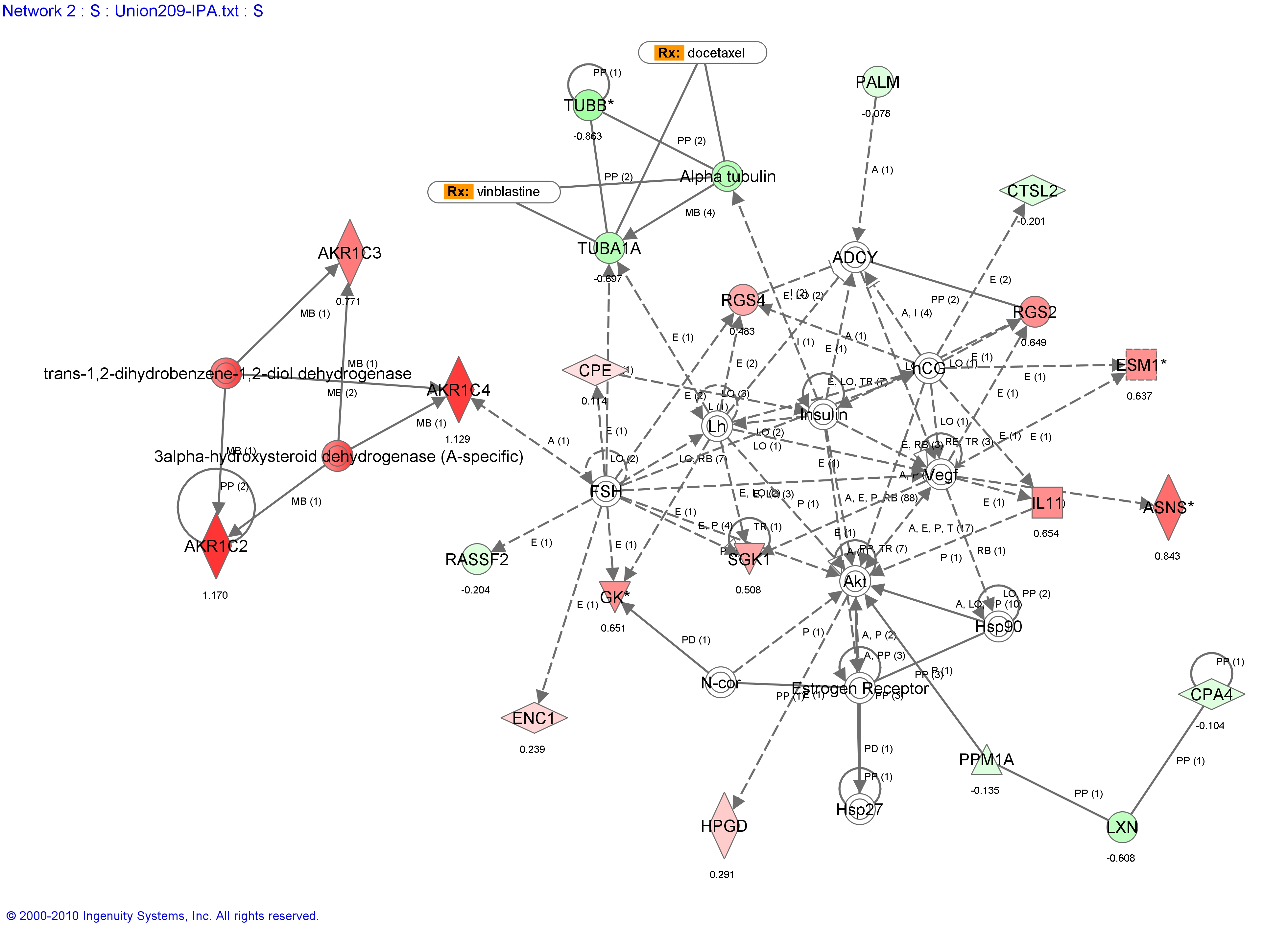

Supplement: Figure S1 — A network enriched by IPA analysis with drug targets (TUBA) of docetaxel and vinblastine (colored in orange) indicated. The union of differentially expressed genes (DEGs) at 24 h and 72 h post CAPE treatment was input to IPA. Upregulated genes are colored in red, and downregulated genes in green. Values of log ratio of expression change were also shown in the bottom of DEGs. (JPG) [file pone.0031286.s001.jpg]

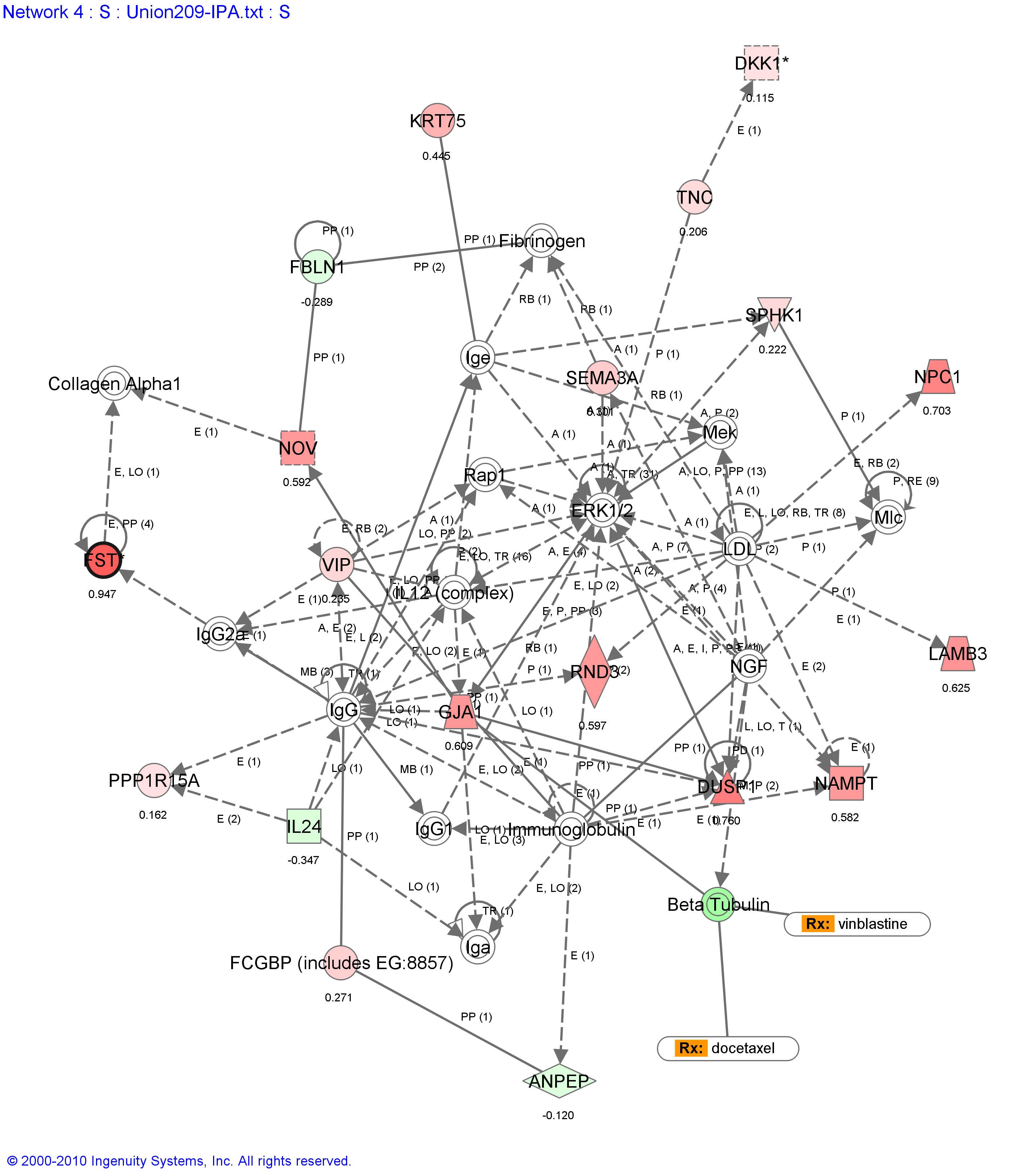

Supplement: Figure S2 — A network enriched by IPA analysis with drug targets (beta tublin) of docetaxel and vinblastine (colored in orange) indicated. The input of IPA analysis and its display is the same as in Figure S1. (JPG) [file pone.0031286.s002.jpg]

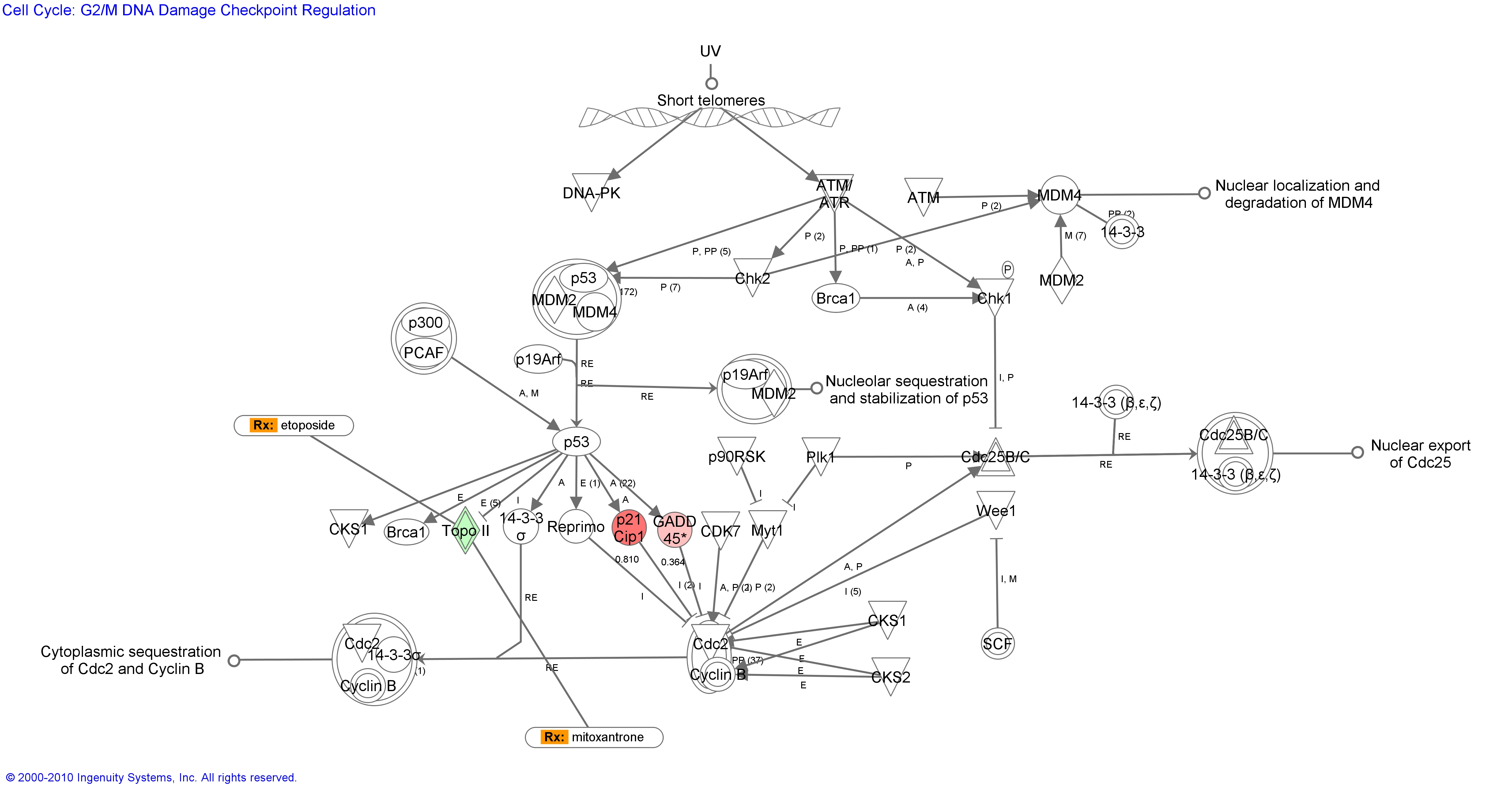

Supplement: Figure S3 — A canonical pathway (G2/M DNA damage checkpoint regulation) enriched by IPA analysis with drug targets (Topo II) of etoposide and mitoxantrone (colored in orange) indicated. The input of IPA analysis and its display is the same as in Figure S1. (JPG) [file pone.0031286.s003.jpg]
